# Supplementary material for: Changes in the Proteome of Platelets from Patients with Critical Progression of COVID-19
Source: Cells. 2023 Sep 1;12(17):2191. doi: 10.3390/cells12172191 (PMC10486756; doi:10.3390/cells12172191)
Supplement: Supplementary file 1 [file cells-12-02191-s001.zip › Supplementary Files-cells-2533000_revised/Supplementary Figures-cells-2533000.pdf]

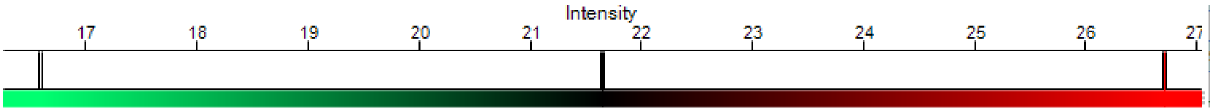


HeatMap Legend

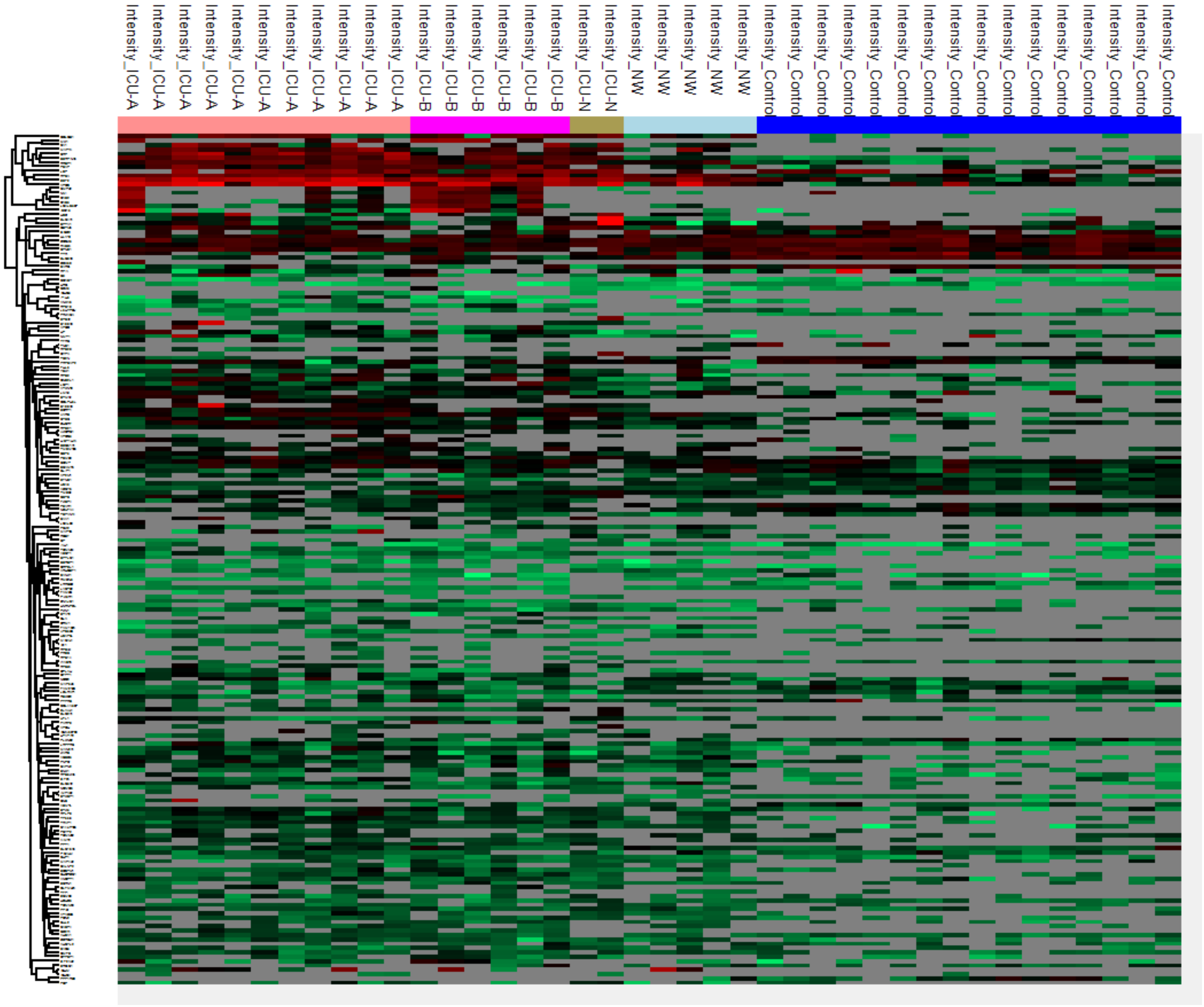


Figure S1: HeatMap: all candidates with missing values. Isolated hierarchical heat map based on the raw intensities of protein groups with at least one missing value in the dataset, showing different levels for the patient groups. Missing values for protein groups are shown in grey. ICU: intensive care unit (ICU A: non-survivors, ICU B: survivors, ICU N: unknown), NW: normal ward.

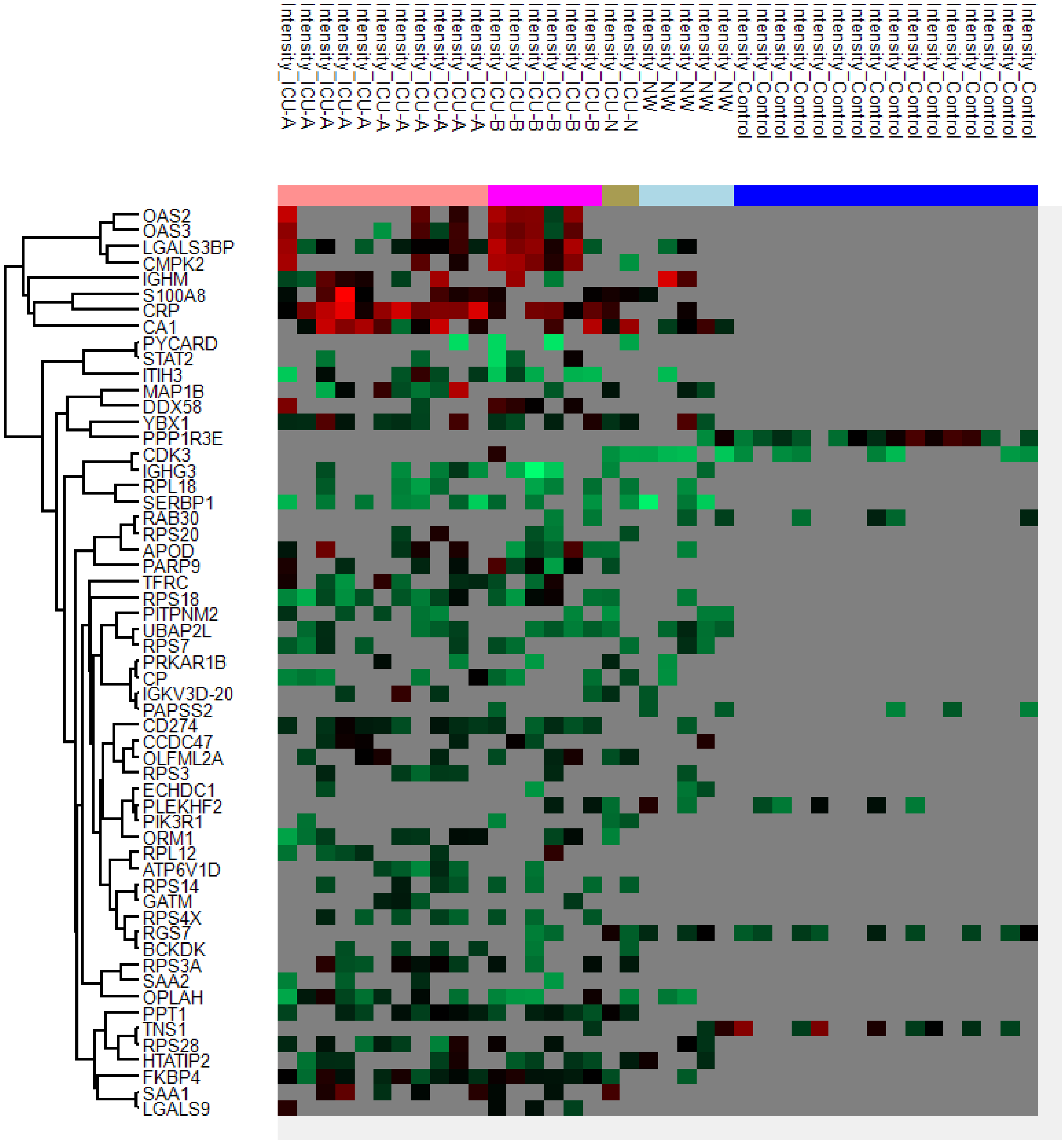


Figure S2: HeatMap: unique proteins. Isolated hierarchical heat map based on the raw intensities of protein groups uniquely identified in one of the following patient groups: controls & NW, controls & NW & ICU B, ICU A & ICU B, ICU A & ICU B & NW. Found in at least 4 samples of the corresponding group. Missing values for protein groups are shown in grey. ICU: intensive care unit (ICU A: non-survivors, ICU B: survivors, ICU N: unknown), NW: normal ward.

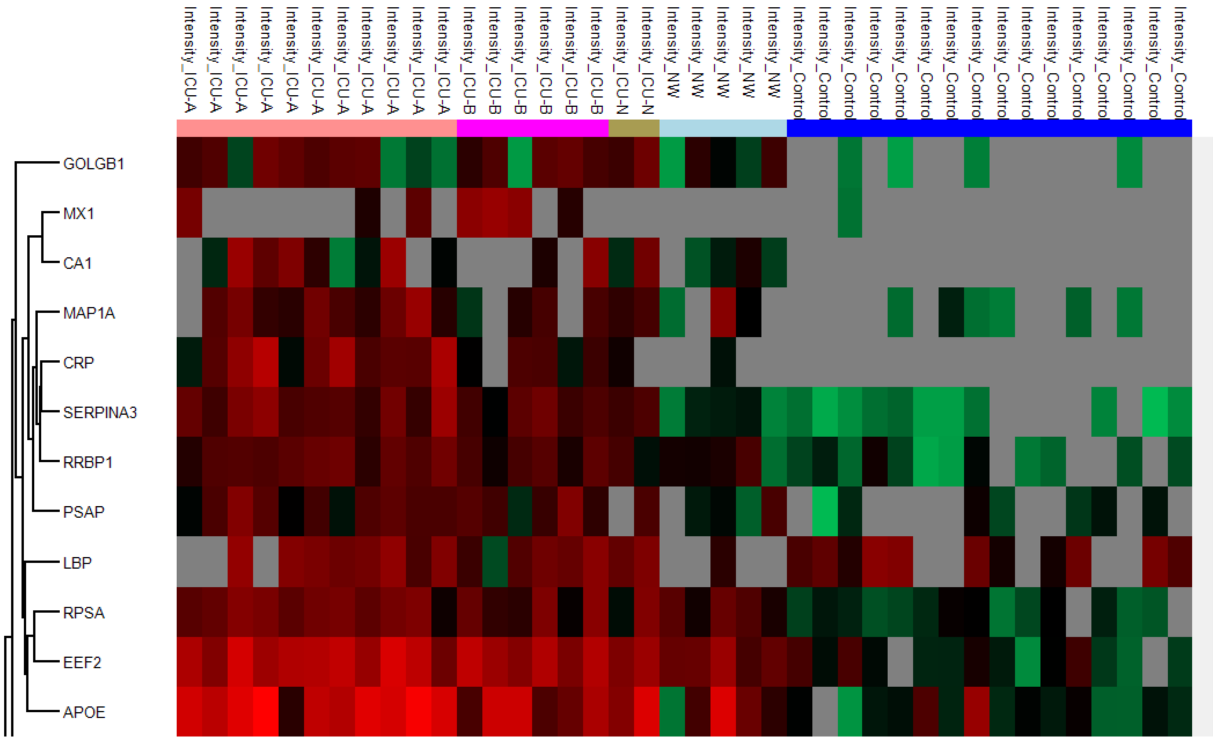


Figure S3: HeatMap: cluster candidates with missing values. Selected cluster from the Fig. S1, showing the most abundant proteins with missing values, found in patients in higher levels than in controls. Missing values for protein groups are shown in grey. ICU: intensive care unit (ICU A: non-survivors, ICU B: survivors, ICU N: unknown), NW: normal ward.
